# Effects of PEG1000 and Sol Concentration on the Structural and Optical Properties of Sol–Gel ZnO Porous Thin Films

**DOI:** 10.3390/ma11101840

**Published:** 2018-09-27

**Authors:** Dong Xu, Qian Yu, Taiyun Chen, Sujuan Zhong, Jia Ma, Li Bao, Lei Zhang, Feiwen Zhao, Sanming Du

**Affiliations:** 1 School of Materials Science and Engineering, Anhui University of Technology, Ma’anshan 243002, China; 2 School of Material Science and Engineering, Jiangsu University, Zhenjiang 212013, China; yuqian0403@126.com (Q.Y.); ctywales@163.com (T.C.); 3 Zhengzhou Research Institute of Mechanical Engineering, Zhengzhou 450001, China; zhongsj@zrime.com.cn (S.Z.); maj@zrime.com.cn (J.M.); baol@zrime.com.cn (L.B.); Zhangl@zrime.com.cn (L.Z.); 4 Jiangsu Xingyuan Power Plant & Melallurglcal Equlpmenl Manuiacluring Co. Ltd., Jingjiang 214500, China; zhaofeiwen@126.com; 5 School of Materials Science and Engineering, Henan University of Science and Technology, Luoyang 471000, China; dsming@haust.edu.cn

**Keywords:** sol–gel method, zinc oxide, porous film, pore formation mechanism, sol concentration

## Abstract

ZnO porous thin films were synthesized as antireflection coatings via a sol–gel dip-coating method with polyethylene glycol (PEG1000) utilized as a polymeric porogen on alumina transparent ceramics. The pore formation mechanism of the ZnO porous thin films was proposed through thermal and Fourier transformation infrared spectrometer (FTIR) analyses. The effect of sol concentrations on crystal structure, microstructure, and optical properties was also discussed. The experiment results indicated that all the ZnO thin films exhibited a hexagonal wurtzite structure with their preferred orientation along a (0 0 2) plane by X-ray diffraction (XRD) patterns. The grain size of the films increased from 30.5 to 37.4 nm with the sol concentration ranging from 0.2 to 1.0 M. Furthermore, scanning electron microscopy (SEM) images show that the pores on the surface were observed to first decrease as the sol concentration increased and then to disappear as the sol concentration continued to increase. The UV spectrum presents a maximum transmittance of 93.5% at a wavelength of 600 nm at a concentration of 0.6 M, which will be helpful in the practical applications of ZnO porous film on alumina transparent ceramic substrates. The pore formation mechanism of ZnO porous thin films can be ascribed to ring-like network structures between the PEG1000 and zinc oligomers under the phase separation effect.

## 1. Introduction

Antireflection (AR) coatings have many great applications in optoelectronic devices that require maximum light transmission such as solar cells and camera lenses [1,2]. They can obviously reduce incident light reflection of the material surface and increase transmission. The high reflectance of alumina transparent ceramics (15~20%) is still a challenging problem which limits the luminous efficiency of sodium vapor lamps. Therefore, searching for appropriate materials that can be used as antireflection coatings on ceramics is an efficient way to improve light transmittance. 

In recent years, many studies have reported the excellent properties of zinc oxide (ZnO) materials [3,4,5,6]. ZnO is a prospective and attractive material for many applications because it is a semiconducting material with a wide and direct band gap (3.37 eV) as well as a large exciton binding energy (~60 meV) [7]. In addition to its transparency in the visible range, the thermal stability of ZnO thin films could help in practical applications such as optoelectronic devices [8], transparent electrodes [9], and electron transport layers [10]. Their high permeability, high refractive index, and strong adherence make ZnO thin films a good antireflective candidate material [11]. Currently, more unique behaviors of ZnO thin films are being explored. Further understanding of the microstructure and optical behaviors of ZnO thin films is vital to its application as an antireflection coating. Due to the wide fields of its applications and novel properties, people have tried different methods to synthesize ZnO thin films. The spray pyrolysis method [12], magnetron sputtering [13], metal oxide chemical vapor deposition (MOCVD) [14], and the sol–gel process [15,16,17] have been used to fabricate ZnO thin films. Among these methods, the sol–gel process is widely adopted for the fabrication of ZnO thin films due to the high speed of the process, its low cost, the possibility of continuous production, the low temperature at which it is processed, and its appropriateness for forming porous ZnO films with an adjustable refractive index.

For an ideal homogeneous single-layer antireflective coating, it is known that the refractive index should satisfy the criterion: n_c_ = (n_0_n_s_)^1/2^, where n_c_, n_0_, and n_s_ represent the refractive indices of the coating, air, and substrate, respectively [18]. The refractive index of alumina transparent ceramics is roughly 1.78, which implies an ideal refractive index of 1.33 for an AR coating since the lowest refractive index for a dense film material is 1.39 [19]. Consequently, porous materials are a better choice for application requirements. Revenant et al. [20,21] have reported that porous In–Ga–Zn–O thin films can be obtained by the rapid quenching of films, where pores can be produced by the evaporation of organics and phase separation on self-organization in the sol–gel process. Unfortunately, this method means that it is difficult to control the structure of the pores and the amount of pores. Therefore, it is necessary to further develop a way to obtain high-quality ZnO porous thin films. Liu et al. [22,23] pointed out that porous ZnO thin films can be produced by using templating and that a high-quality porous structure can be obtained. So far, many porous nanostructures have been reported when polyethylene glycol (PEG1000) was used as the polymeric porogen. PEG1000 in the SiO_2_ sols can form networks with ring-like structures in short-order due to the linkage of PEG1000 with sol particles [24,25]. However, there are only a few studies in the literature investigating ZnO porous thin films with PEG1000.

The aim of the present work was to prepare ZnO porous thin films on alumina transparent ceramics, which can be used as antireflection coatings to improve the light transmittance of devices. Chemical and physical changes during the sol–gel process were analyzed. The pore formation mechanism of ZnO porous thin films was discussed. In addition, we investigated the effects of sol concentration on the structure, surface morphology, and optical properties of ZnO porous thin films deposited on alumina transparent ceramics substrates using a sol–gel dip-coating method.

## 2. Experimental Details

### 2.1. Materials

ZnO porous thin films were fabricated on 50 × 8 × 1 mm^3^ alumina transparent ceramic substrates (Changzhou Mingde Ceramics Co. Ltd., Changzhou, China) using the sol–gel method. In the sols, zinc acetate dihydrate (Zn(CH_3_COO)_2_·2H_2_O, Sinopharm, China) was utilized as the precursor. Ethanol (CH_3_CH_2_OH, Sinopharm, China), diethanolamine (DEA, NH(C_2_H_2_OH)_2_, Sinopharm, China), and a polyethylene glycol (PEG1000, (C_2_H_4_O)_n_H_2_O, Sinopharm, China) solution were used as the solute, the solvent, and the stabilizer and polymeric porogen, respectively. All of the chemicals were used without further purification.

### 2.2. Sol Preparation and Deposition of Films

**Figure 1:**
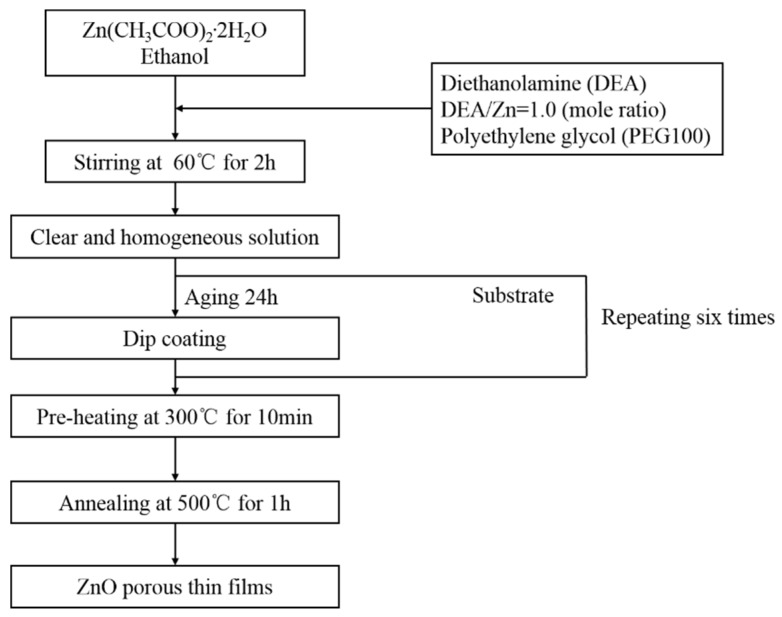
The flow chart showing the procedure for preparing the ZnO porous thin films.

First, the zinc acetate dihydrate was poured into ethanol and then stirred by a magnetic stirrer at a constant temperature of 60 °C to be mixed fully. After 10 min, diethanolamine (DEA) was dropped into the mixed solution within 30 s. The molar ratio of DEA:zinc acetate was always 1.0:0. Then, 1.0 g of polyethylene glycol (PEG1000) was added to the solution as a polymeric porogen. In the solutions, the concentrations of Zn^2+^ were 0.2 M, 0.4 M, 0.6 M, 0.8 M, and 1.0 M, respectively. A clear and homogeneous solution was obtained after stirring for 2 h at 60 °C. Finally, the homogeneous solution was baked for 24 h. The substrates were washed successively with acetone, methanol, and deionized water. ZnO porous thin films of different sol concentrations were dip-coated on the cleaned substrates with a drawing speed of 6 cm/min. While dipping, we maintained 60 s for residence time to ensure that the film could be coated onto the substrate. After this procedure, the films were directly preheated at 300 °C for 10 min so as to steam the solvent and remove any organic substances. The processes, from dip-coating to preheating treatment, were repeated six times so that the film reached the desired thickness. Finally, the substrates were heated at 500 °C for 1 h using heating rates of 2 °C/min in air to obtain ZnO porous thin films. The procedure used for preparing the ZnO thin films is shown in Figure 1. 

### 2.3. Characterization 

The Thermogravimetric (TG) and Differential Scanning Calorimetry (DSC) analyses of the ZnO xerogel were characterized by a thermal analyzer (STA449C) with a heating rate of 10 °C/min in air. FTIR spectra of the sol were measured by a Fourier transformation infrared spectrometer (Nicolet iS50, Thermo Fisher Scientific, Waltham, MA, USA) in transmission mode using the principle of optical interference whereby the interference light carrying the sample information is accepted by the detector and the data are finally transmitted for signal processing. The crystal structures of the films were confirmed by X-ray diffraction (XRD, Japanese science D/MAX2500PC) using Cu Kα radiation (λ = 1.54178 Å) operated at 40 kV, 40 mA. MFP-3D scanning electron microscopy (SEM, JSM-7001F, Nidec Corporation, Tokyo, Japan) was performed to reveal the surface morphology. The transmittance spectra were measured using an UV–Vis spectrophotometer (Schimadzu UV-2550). 

## 3. Results and Discussion

### 3.1. Mechanism of Pore Formation

**Figure 2:**
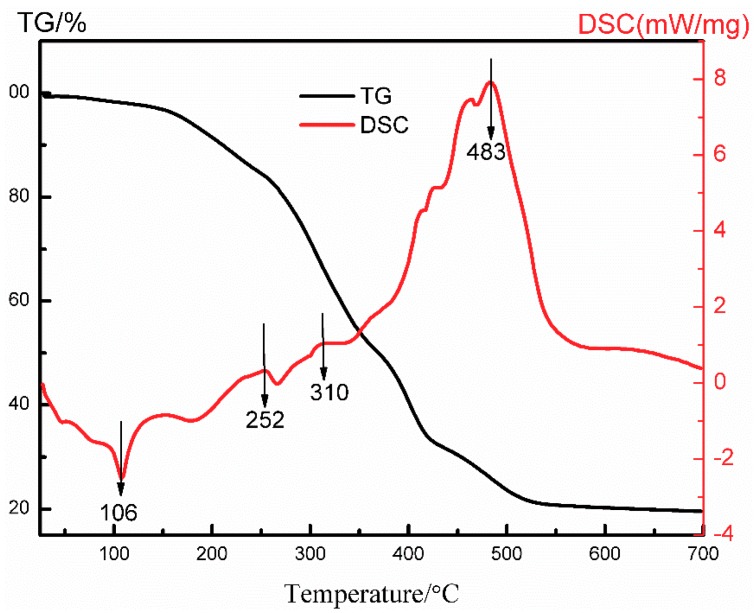
TG–DSC curve of the ZnO xerogel.

Figure 2 depicts the thermal behaviors of the ZnO xerogel using a thermal analyzer in air at a heating rate of 10 °C/min. Four-step weight losses were found in the TG curve corresponding to 25–150 °C, 150–420 °C, 420–500 °C, and 500–700 °C, respectively. About 3.3% of the first-step weight loss was assigned to the evaporation of ethanol and water. The second-step weight loss was about 63.3% and originated from the disintegration and the inflammation of the DEA and the PEG1000. In the third step, the combustion of the remnants was the main reason for the weight loss of 12.6%. Finally, it was found that no weight loss appeared above 500 °C. One endothermic peak at 106 °C and three exothermic peaks at 252 °C, 310 °C, and 483 °C in the DTA curve corresponded to the above four weight losses, respectively. There was an especially huge exothermic peak at 483 °C, which may have led to the crystal transformation of ZnO from an amorphous state to a wurtzite phase. The results show that an annealing temperature of 500 °C would be suitable for fabricating ZnO thin films. 

**Figure 3:**
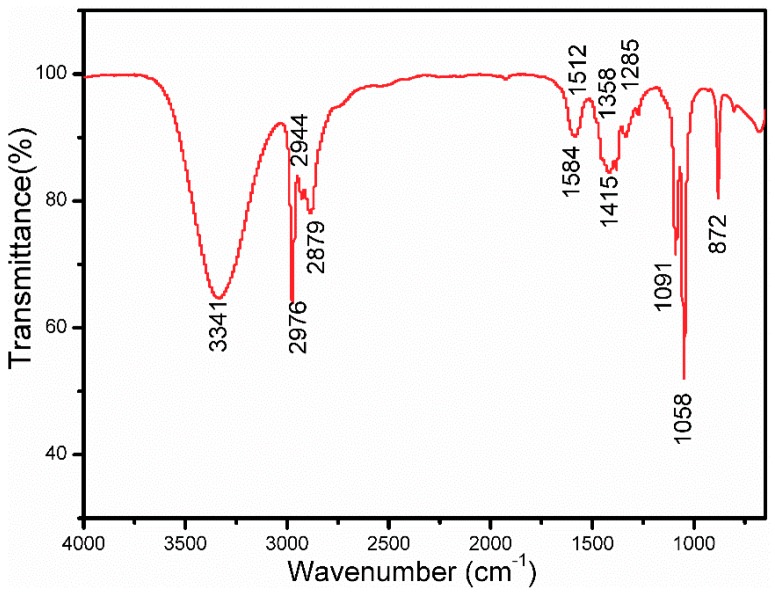
FTIR spectra of the ZnO sol with PEG1000.

The FTIR spectra of the ZnO sol were measured to explore the chemical changes of the sol–gel process. As depicted in Figure 3, during the process, the vibration at 3341 cm^−1^ is ascribable to the O–H stretching vibration of H_2_O, which is formed through polycondensation. The peak at 2976 cm^−1^ is attributable to the N–H group and the small absorption peaks at 2944 cm^−1^ and 2879 cm^−1^ are attributable to the C–H asymmetric and symmetrical vibrations of the DEA, respectively. Furthermore, the peaks at 1584 cm^−1^ and 1415 cm^−1^ are attributable to the C=O asymmetric and symmetrical stretching vibrations. It also indicated that the DEA and the zinc acetate formed complex compounds in the sol which promote the synthesis of a clear and homogeneous solution. Peaks at 1058 cm^−1^ and 1091 cm^−1^ are ascribable to the C–N stretching of the DEA. There are two small vibrations at 1358 cm^−1^ and 1285 cm^−1^ due to the –CH_2_–O–Zn stretching vibration, which is synthesized by PEG1000 and zinc oligomers. Moreover, the peak at 872 cm^−1^ is the result of the C–H out-of-plane bending vibration, which indicates that the PEG1000 and zinc oligomers formed a network structure through their strong interaction. Therefore, from the chemical changes during the sol–gel process, it may be deduced that zinc acetate is firstly complexed with DEA, zinc oligomers are then produced, and PEG1000 finally interacts with the zinc oligomers to form a network structure. The pore formation mechanism of ZnO porous thin films is discussed in detail as follows. 

**Figure 4:**
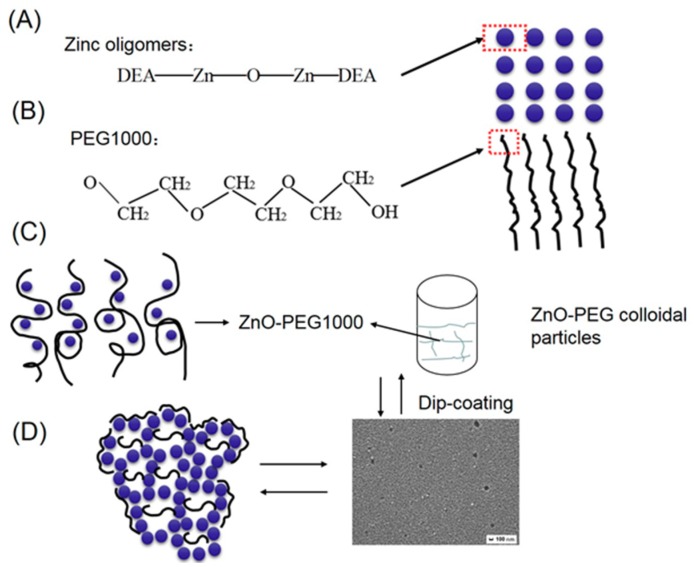
(**A**) Schematic representation of the zinc sols, (**B**) PEG1000, (**C**) PEG1000 connected with sol particles, and (**D**) porous ZnO thin films.

It has been suggested that PEG1000 affects the zinc acetate dihydrate system by hydration and through its interactions with ZnO particles, including chemical and hydrogen bonding [23,26]. Moreover, based on the schematic model for the formation of TiO_2_ films [24,27], the schematic model for the formation of ZnO porous thin films is shown in Figure 4. As demonstrated in Figure 4A, DEA complexed with [CH3COOZn]^+^ and those complex compounds can prevent the fast hydrolysis of sol particles. During the process, zinc oligomers can be formed. Figure 4B shows that the PEG1000 molecule is a long zigzag chain structure. The structures consisting of –O– and –CH2–CH2– are hydrophilic and hydrophobic, respectively. In Figure 4C, when PEG1000 was added to the sol, it initially interacted chemically with the sol particles to produce ZnO–PEG1000 colloidal particles. Meanwhile, the sol began to form pore structures due to the phase separation effect [24]. Those particles then formed ring-like network structures because of the strong interaction between the PEG1000 and the zinc oligomers under appropriate conditions. As shown in Figure 4D, after the dip-coating process, the final pores of the thin films can be produced by the volatilization, decomposition, and combustion of the ethanol solvent and the PEG1000 molecules. SEM images demonstrated this structure. As shown in the SEM images, ZnO porous thin films were successfully fabricated with PEG1000 as a polymeric porogen. Moreover, it can be clearly seen that the pores are distributed uniformly in the film in a short order.

### 3.2. Characteristics of the ZnO Porous Thin Film

**Figure 5:**
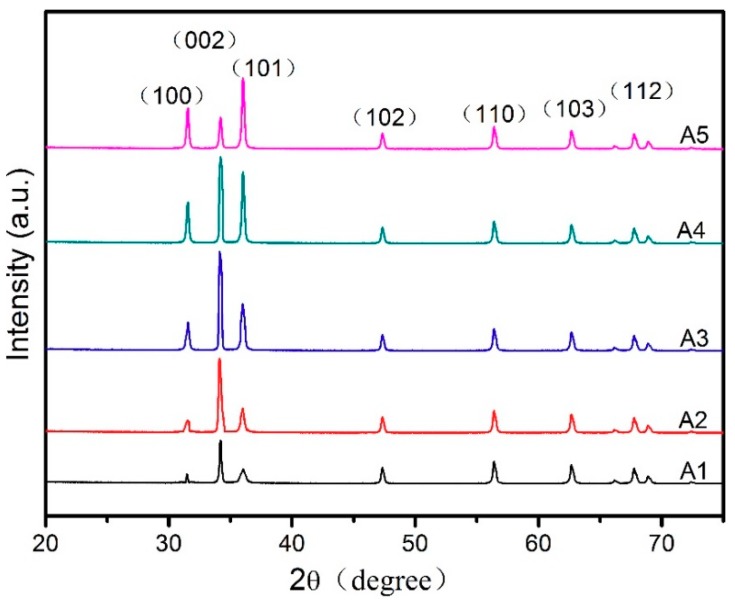
X-ray diffraction spectra of the ZnO thin films for sol concentrations of (**A1**) 0.2 M, (**A2**) 0.4 M, (**A3**) 0.6 M, (**A4**) 0.8 M, and (**A5**) 1.0 M.

Figure 5 illustrates the XRD patterns of the ZnO porous thin films fabricated at different sol concentrations. All of the diffraction peaks of the designs correspond to a reflection of the wurtzite-structured ZnO planes and all of the ZnO porous films possess a strong (0 0 2) peak, except for A5. It is widely believed that the prepared films possess a hexagonal wurtzite structure and are preferentially orientated along the c-axis perpendicular to the substrate surface [28,29,30]. Also, it can be seen that the intensity of the (0 0 2) peak increases as the sol concentration increases to 0.6 M and then decreases from 0.8 M to 1.0 M, thus indicating a change in the film’s microstructure.

**Figure 6:**
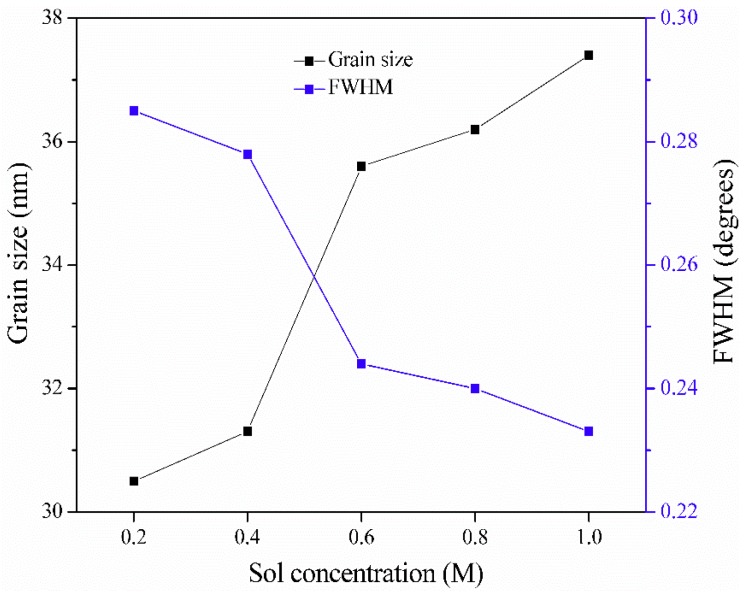
Variation of the grain size and the full width at half maximum intensity (FWHM) of the ZnO films according to the sol concentration.

Table 1 shows the detailed full width at half maximum intensity (FWHM), the interplanar spacing (d), and the grain size (*D*). The average grain size was deduced from the XRD patterns using the Debye–Scherrer formula [31].

(1) $$D=\frac{0.94\lambda }{\Delta \left(2\theta \right)cos\theta }$$

where *D* is the grain size, *λ* = 0.154 nm represents the X-ray wavelength, 2*θ* is the diffraction angle corresponding to the (0 0 2) plane, and ∆ (2*θ*) is the full width at half maximum intensity (FWHM) of the peak. As the ZnO precursor concentration increased from 0.2 to 1.0 M, the mean grain size showed a gradual increase from 30.5 to 37.4 nm. As shown in Table 1, the 2*θ* value of the (0 0 2) plane increases slightly in all of the samples as the concentration increases. In addition, as the sol concentration increases, the interplanar spacing (d) of the films exhibits a tendency to decrease that is relevant to the gradual increase of the grain size. As shown in Figure 6, the FWHM value decreases almost linearly as the sol concentration increases, indicating the better crystallinity of films with higher sol concentrations. The increase of the grain size also means that the crystallinity becomes better as the sol concentration increases [32]. This result is also demonstrated in the SEM images.

**Figure 7:**
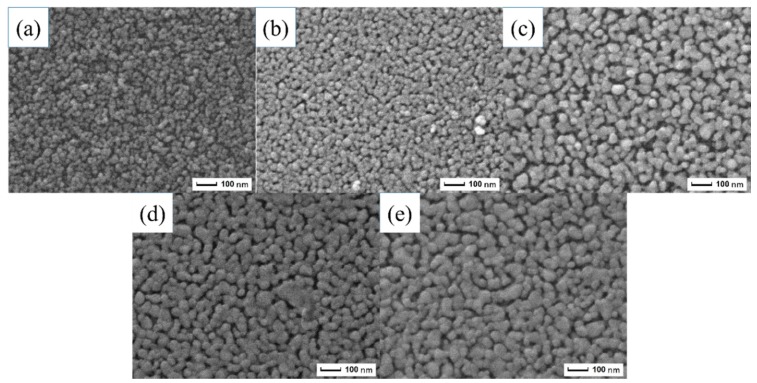
Scanning electron microscopy (SEM) images of the ZnO films at different sol concentrations: (**a**) 0.2 M, (**b**) 0.4 M, (**c**) 0.6 M, (**d**) 0.8 M, and (**e**) 1.0 M.

In order to reveal the influence of sol concentrations on the surface morphology, SEM images of the ZnO porous thin films were captured, as presented in Figure 7. In Figure 7a, it can be observed that the ZnO thin films consist of aggregates of round-shaped particles and some cracks. This means that the films cover the substrate in a rough and inhomogeneous fashion at low sol concentrations. In Figure 7b, the films become more uniform and begin to show a micropore structure. The formation of films with a micropore structure is quite obvious in Figure 7c. The surface morphology of this film indicates that the PEG1000 perfectly connected with the zinc oligomers and could lead to the appearance of pores under phase separation effects. The ZnO single layer can be treated as a homogeneous coating with a uniform refractive index. However, as shown in Figure 7d–e, the micropore structure began to disappear. This change can be explained by the variation in the sol concentration. As the concentration increases, the gradually increasing volume of solute (i.e., zinc acetate) in the sol makes the electrostatic interaction between the solute particles greater. Thereby, this phenomenon increases the probability that more solute would gather together to form a grain and thus decrease the amount of micropore structures in the films.

**Figure 8:**
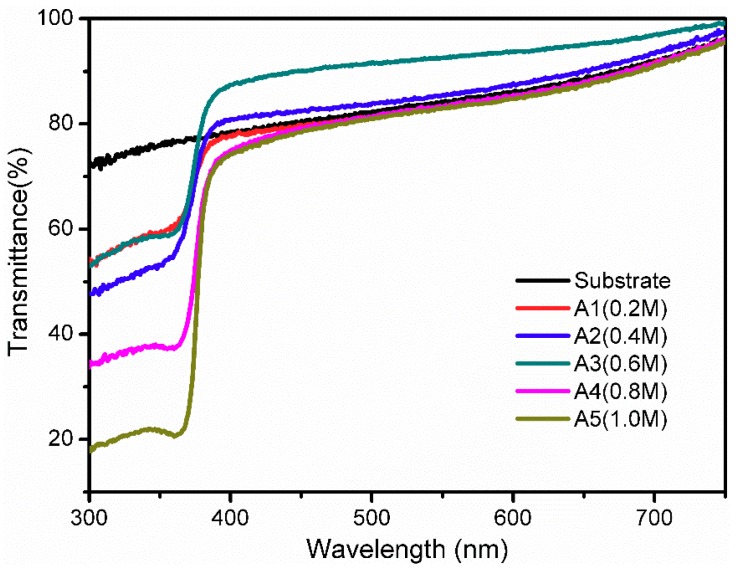
Transmittance spectra of the ZnO films deposited with different sol concentrations.

To further explore the effects of sol concentration on the optical transmittance of ZnO porous thin films, the optical transmittance and the band gap (*E*_g_) were discussed. The optical transmittance of ZnO porous thin films fabricated at different sol concentrations is shown in Figure 8. All of the films are highly transparent throughout the visible range, with transmittance values of more than 75%. As can be seen from the spectra, the films coated with 0.4 M and 0.6 M sol concentrations have higher transmittance values than the alumina transparent ceramic substrates. Specifically, the highest transmittance of the ZnO porous thin films increased by approximately 8.5% compared with the substrates in the visible range. Therefore, these films can be treated as antireflection coatings [33,34,35]. However, the films fabricated with 0.2 M, 0.8 M, and 1.0 M sol concentrations showed lower transmittance values than the substrates. Surface morphology has a significant influence on optical properties. This change in transmittance might be because of the appearance of the micropore structure in the porous films, as discussed earlier when considering the SEM images. The decrease in the transmittance is also attributable to optical scattering caused by some cracks and the increased grain size in the porous films.

**Figure 9:**
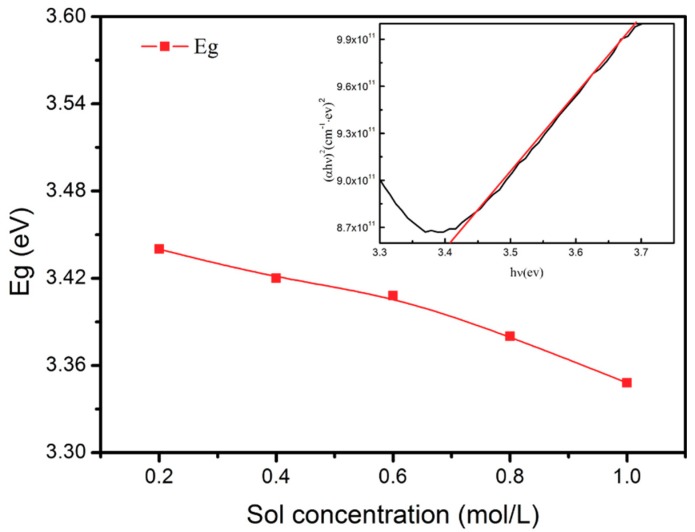
Variation of band gaps of ZnO films according to the sol concentration. Inset: typical plot of (αhυ)^2^ versus photon energy (*hυ*) of the 0.6 M ZnO films.

In the optical transmittance spectra, the transmittance increased sharply for all of the samples near the ultraviolet region. This phenomenon can be explained by the intrinsic band gap of ZnO [36,37,38]. Figure 9 shows the variety of optical band gaps of the ZnO films at different sol concentrations. The formula for the optical absorption edge is as follows [39]:

(2) $$\left(\alpha h\upsilon \right)=A{\left(h\upsilon -E_{g}\right)}^{n}$$

where *α* is the absorbance value, *hυ* = 1240/*λ* is the photon energy of incident light, *A* is a constant, and *n* is equal to 1/2 for direct band gaps. The optical band gap (*E*_g_) was evaluated via the (*αhυ*)^2^ vs. (*hυ*) relationship by extrapolating the linear region of the curves on the energy axis ((*αhυ*)^2^ = 0). As shown in Figure 9, the optical band gap of the ZnO films reduces from 3.44 to 3.35 eV as the sol concentration increases from 0.2 to 1.0 M. This result is consistent with previous studies reported by M. Salem [12]. The reduction of band gap energy may be due to an increase in the grain size and a minimization of defects on the surface.

## 4. Conclusions

ZnO porous thin films were successfully synthesized on alumina transparent ceramic substrates via the sol–gel method utilizing polyethylene glycol (PEG1000) as a polymeric porogen. Through thermal and FTIR studies, it was confirmed that the pore formation mechanism of ZnO porous thin films is the formation of ring-like network structures from connections between PEG1000 and zinc oligomers under the phase separation effect. It was found that the sol concentration has a critical effect on the crystal structure, microstructure, and optical properties of ZnO thin films. The sample prepared with 0.6 M sol showed the best preferential orientation along the c-axis in the XRD pattern. From SEM images, an obvious pore network structure can be observed on the films’ surface at 0.6 M sol concentration. Studies on their optical properties showed that the ZnO porous films at 0.6 M sol achieve the highest transmittance in the visible range and that the optical band gap was reduced with an increase of sol concentration from 0.2 M to 1.0 M. 

## Figures and Tables

**Table 1:** The structural parameters of the (0 0 2) plane of the ZnO porous thin films deposited at different sol concentrations (2*θ*: peak position, d: interplanar spacing, and *D*: grain size.).

Sol Concentration (M)	2*θ* (002) (°)	FWHM (°)	d (nm)	*D* (nm)
0.2	34.217	0.285	0.26184	30.5
0.4	34.278	0.278	0.26094	31.3
0.6	34.338	0.244	0.26006	35.6
0.8	34.398	0.240	0.25962	36.2
1.0	34.428	0.233	0.25874	37.4
